# Protein expression under sustained activation of signal transducer and activator of transcription-3 in diethylnitrosamine-induced rat liver carcinogenesis

**DOI:** 10.3892/ol.2014.2194

**Published:** 2014-05-28

**Authors:** LI-MIN CUI, KUN ZHANG, DONG-JIE MA, SHUANG-PING LIU, XUE-WU ZHANG

**Affiliations:** 1 Department of Biochemistry and Molecular Biology, College of Medicine, Yanbian University, Yanji, Jilin 133002, P.R. China; 2 Department of Surgery, Affiliated Hospital, Yanbian University, Yanji, Jilin 133002, P.R. China

**Keywords:** signal transducer and activator of transcription-3, sustained activation, diethylnitrosamine, rats, liver cancer

## Abstract

The aim of the present study was to investigate the expression of proteins associated with the sustained activation of the signal transducer and activator of transcription (STAT)-3 pathway during diethylnitrosamine (DEN)-induced rat liver carcinogenesis. DEN was intermittently administered to rats to induce liver cancer, and light and electron microscopy were used to observe the morphological changes in the liver during carcinogenesis. Western blotting and quantitative polymerase chain reaction (qPCR) were used to detect the expression of STAT-3, phosphorylated (p)-STAT-3, matrix metalloproteinase (MMP)-10, vascular endothelial growth factor (VEGF), kinase insert domain receptor (KDR), hypoxia inducible factor (HIF)-1α, basic fibroblast growth factor (bFGF) and interleukin (IL)-10, in order to investigate the association between STAT-3 and p-STAT-3 expression and MMP-10, VEGF, KDR, HIF-1α, bFGF and IL-10. The western blotting and qPCR results revealed that the expression of STAT-3, p-STAT-3, MMP-10, VEGF, KDR, HIF-1α, bFGF and IL-10 proteins gradually increased during carcinogenesis. Furthermore, the STAT-3 and p-STAT-3 levels were found to positively correlate with MMP-10, VEGF, KDR, HIF-1α, bFGF and IL-10 protein expression. During DEN-induced rat liver carcinogenesis, STAT-3 protein continually activated MMP-10, VEGF, KDR, HIF-1α, bFGF and IL-10, and its expression was found to positively correlate with the expression of these proteins.

## Introduction

Tumor invasion and metastasis is a complex process in which tumor cells lose their cell-cell adhesion, penetrate the basement membrane and extracellular matrix (ECM) at the primary site to enter the blood circulation, and then evade immune surveillance and migrate to other areas of the body to continue growing (1). When cellular signal transduction is normal, signal transducer and activator of transcription (STAT)-3 is under multi-level control by multiple factors, its signaling strength and dynamics maintain specificity (2) and its activation is rapid and transient. In tumor cells, there is continuous STAT-3 activation, resulting in disordered janus tyrosine kinase (JAK)-STAT signal transduction, which is typical of tumor cells during invasion and metastasis (3). STAT-3 may be activated by a variety of cytokines, in particular, those in the interleukin (IL)-6 family, such as gp130 (4). Jenkins *et al* (5) found that STAT-3 deletion mutants completely reversed the splenomegaly, hepatic acute phase reaction, abnormal lymphocyte activation and spontaneous gastric antrum cancer observed in gp130 mutant mice, demonstrating that the sustained activation of STAT-3 is important for the abnormal proliferation of a variety of cells. Haura *et al* (6) showed that the expression of the STAT-3 mutant, STAT-3-C (with cysteine substitutions at amino acids at A661 and N663), is carcinogenic, further confirming that the sustained activation of STAT-3 leads to cell transformation, which is closely associated with human carcinogenesis. In the present study, the diethylnitrosamine (DEN)-induced rat liver cancer model was used to simulate the induction and development of human liver cancer. The expression of STAT-3 was observed and the correlation between tumor metastasis, invasion, angiogenesis and immune escape, and the expression of the matrix metalloproteinase (MMP)-10, vascular endothelial growth factor (VEGF), kinase insert domain receptor (KDR), hypoxia inducible factor (HIF)-1α, basic fibroblast growth factor (bFGF) and IL-10 proteins was investigated. Elucidating the mechanisms of liver carcinogenesis and progression may contribute to the prevention of this disease and the development of targeted therapy.

## Material and methods

### Rat liver cancer model

A total of 136 male five-week-old Wistar rats [SCXK-(Ji) 2007-0003; Experimental Animal Center of Bethune Medical College of Jilin University, Certificate of Conformity, Yanji, Jilin, China], weighing of 140–160 g, which had been fed stably for seven days, were divided into experimental and control groups. Sterile drinking water containing 0.01% DEN (purity 99.9%; Sigma-Aldrich, St. Louis, MO, USA) was provided *ad libitum* to the experimental group (n=120). This was replaced every day. After five weeks, with DEN-free water was provided for three weeks, followed by 0.01% DEN solution for 12 weeks and then complete withdrawal of the drug. DEN-free sterilized drinking water was provided to the control group (n=16) for the entire study duration. A total of 15 experimental rats were sacrificed at 4, 8, 12, 16, 18 and 20 weeks post-treatment, respectively, with two control rats of the same age also sacrificed at each time-point. The study was approved by the ethics committee of Yanbian University (Yanji, China).

### Specimen collection and processing

The appearance, color and texture of the rat livers were recorded. Certain sections of the liver or liver cancer tissues were fixed in 4% paraformaldehyde (Jinzhou Chemical Reagent Plant, Dalian, China), paraffin-embedded and sectioned for hematoxylin and eosin staining (Shengyang Chemical Reagents Factory, Shenyang, China). Additional sections of the liver or the liver cancer tissues (1×1×1 mm) were fixed in 2.5% glutaraldehyde (Shengyang Chemical Reagents Factory) at 4°C, rinsed twice in phosphate-buffered saline (0.1 mol/l) and fixed in 1.0% osmium tetroxide (Huaye Chemical Industry Co., Ltd., Beijing, China). The samples were then embedded in EPON812 (Sigma-Aldrich) in order to generate ultra-thin sections, which were double-stained with uranyl acetate (Sigma-Aldrich) and lead citrate, and then observed using a JEM1200EX transmission electron microscope (JEOL, Tokyo, Japan).

### Western blotting

The liver and tumor tissues were lysed in lysis buffer (Pierce Biotechnology, Inc., Rockford, IL, USA) and centrifuged at 12,000 × g for 15 min. The protein concentration was determined using the bicinchoninic acid kit (Pierce Biotechnology, Inc.) according to the manufacturer’s instructions. A 70-μg protein sample was fractionated by 10% sodium dodecyl sulfate polyacrylamide gel electrophoresis and then transferred to a polyvinylidene fluoride membrane (Pall Corporation, Port Washington, NY, USA). Subsequent to blocking the membranes for 1 h with 5% milk in Tris-buffered saline and Tween-20, the primary antibodies, rabbit monoclonal anti-MMP-10, -VEGF, -KDR, -HIF-1α, -bFGF and -IL-10, and rabbit monoclonal anti-STAT-3 (1:400; catalogue number: BA0621, Boshide Biotech Co. Ltd., Wuhan, China) and -phosphorylated (p)-STAT-3 (1:400; Boshide Biotech Co. Ltd., Wuhan, China), were added and incubated at 4°C overnight. Following incubation with goar anti-rabbit IgG peroxidase-labeled secondary antibodies (1:5,000; catalogue number: BA1055, Boshide Biotech Co. Ltd.) the proteins were visualized by chemiluminescence. The intensity of the protein bands was quantitatively determined using an ultraviolet crosslinker (Bio-Rad, Hercules, CA, USA) and normalized with the intensity of the actin band in each gel.

### Quantitative polymerase chain reaction (qPCR)

The RNeasy Plus Mini Kit (Qiagen) was used according to the manufacturer’s instructions to extract total RNA from the tumors. cDNA was generated with the iScript Select cDNA Synthesis kit (Qiagen) and then analyzed by qPCR using SyberGreen qPCR primer assays (Qiagen, Hilden, Germany) and the iCycler iQ Multicolor Real-Time PCR Detection System (Qiagen). The relative expression levels were normalized against β-actin expression, which was run simultaneously as a reference control. The primers used are listed in Table I.

### Statistical analysis

Data are presented as the mean ± standard deviation. One-way analysis of variance and independent t-tests of the sample pairs were used, and all data analyses were performed using SPSS version 13.0 software (SPSS, Inc., Chicago, IL, USA). Bivariate correlation analysis was used to analyze the correlation data. P<0.05 was considered to indicate a statistically significant difference.

## Results

### Rat liver pathology

**Figure 1:**
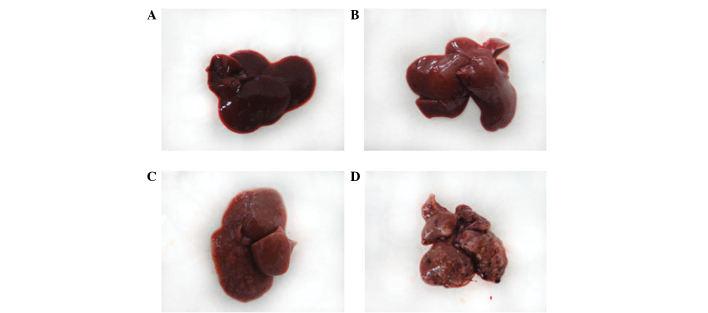
Changes observed in liver tissue during the development of diethylnitrosamine (DEN)-induced rat liver cancer. (A) Normal group, the liver has a smooth, glossy surface and a soft texture. (B) Experimental group (early carcinogenesis; 1–8 weeks), the liver is of normal appearance. (C) Experimental group (interim carcinogenesis; 9–15 weeks), the liver has a rough surface with gray lesions. (D) Experimental group (late carcinogenesis; 16–20 weeks), the surface of the liver is covered with nodules of various sizes.

**Figure 2:**
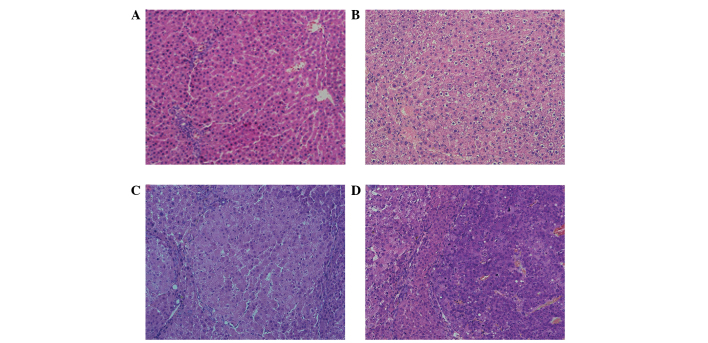
Changes in liver tissue during the development of diethylnitrosamine (DEN)-induced rat liver cancer, as observed by light microscopy (stain, hematoxylin and eosin; magnification, ×200). (A) Normal group, the architecture of the hepatic lobes is complete, with hepatocytes arranged in neat rows and the clear cell nuclei. (B) Experimental group (early carcinogenesis; 1–8 weeks), the architecture of the hepatic lobes is complete, and there is visible intralobular focal necrosis with infiltrating inflammatory cells. (C) Experimental group (interim carcinogenesis: 9–15 weeks), the architecture of the hepatic lobes is damaged and the hepatocytes are proliferating, accompanied by severe steatosis and the formation of visible pseudolobules. (D) Experimental group (late carcinogenesis; 16–20 weeks), the cancer cells exhibit evident atypia, with abnormally large nuclei and diminished cytoplasm.

**Figure 3:**
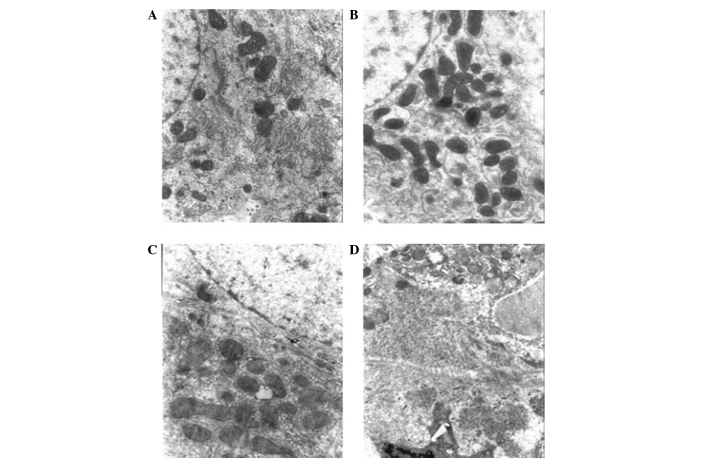
Changes in liver tissue in diethylnitrosamine (DEN)-induced rat liver cancer observed by electron microscopy (magnification, ×6,000). (A) Normal group, the hepatocytes are round or oval and in regular arrays, the ratio of nucleus to cytoplasm is normal and cytoplasmic organelles are abundant. (B) Experimental group (early carcinogenesis; 1–8 weeks), the hepatocytes are swollen, with swollen mitochondria. The granular matrix has disappeared and granulovacuolar degeneration can be observed. (C) Experimental group (interim carcinogenesis; 9–15 weeks), hepatocyte chromatin is aggregated, the number of mitochondria has increased, the cristae of the mitochondria are disrupted, the rough endoplasmic reticulum is dilated, the nuclear membrane is uneven and the nucleolus has moved to the side of the cell. (D) Experimental group (late carcinogenesis; 16–20 weeks), the hepatocyte nuclei are larger, the number of mitochondria is reduced, the structure of the mitochondria is disrupted, the rough endoplasmic reticulum has lost its layered structure, the plasma membrane is fragmented and the ribosomes have separated from the rough endoplasmic reticulum.

The surface of the liver of the control rats was brown, soft in texture and smooth, with an evident gloss (Fig. 1A). Light microscopy revealed that the structure of the hepatic lobule was complete, with hepatocytes arranged in neat rows and with clear nuclei (Fig. 2A). In addition, electron microscopy revealed a regular, rounded or oval cell morphology, and a normal nucleus to cytoplasm ratio. Abundant cytoplasmic organelles were present, and the mitochondria, rough and smooth endoplasmic reticulum and Golgi complex were well developed (Fig. 3A).

The experimental rat liver pathology may be divided into three temporal stages: The early carcinogenesis-hepatocyte injury period at 1–8 weeks, the interim carcinogenesis-sclerosis period at 9–15 weeks and the late carcinogenesis-cancer period at 16–20 weeks. In the early carcinogenesis-hepatocyte injury period, the appearance of the liver was not evidently abnormal (Fig. 1B); when observed by light microscopy, the architecture of the hepatic lobes was complete, however, ballooning degeneration was exhibited by certain cells. Visible intralobular focal necrosis with inflammatory cell infiltration, and gradual emergence of fibrous tissue proliferation and the regeneration of hepatocytes were identified (Fig. 2B). In addition, electron microscopy revealed swelling hepatocytes, with swelling mitochondria and the disappearance of the granular matrix, which was accompanied by granulovacuolar degeneration (Fig. 3B). In the interim carcinogenesis-sclerosis period at 9–15 weeks, the surface of the liver gradually roughened, and varying numbers of large and small gray lesions appeared (Fig. 1C). Furthermore, light microscopy revealed that the normal lobular structure had been destroyed, the hepatocytes had been replaced by fibrous tissue and the typical pseudolobular structure had formed (Fig. 2C). Additionally, electron microscopy revealed aggregated hepatocyte chromatin, an increased number of mitochondria, disrupted mitochondrial cristae, dilated rough endoplasmic reticulum, an uneven nuclear membrane and nucleoli that had moved to the edges of the cells (Fig. 3C). In the late carcinogenesis-cancer period at 16–20 weeks, the surface of the liver was covered with multiple large and small nodules (Fig. 1D). Furthermore, light microscopy revealed that the cancer cells exhibited evident atypia, with larger nuclei and less cytoplasm than normal. A number of monocytes and mitotic figures were also identified (Fig. 2D). Additionally, electron microscopy revealed hepatocyte nuclei of increased size, decreased numbers of mitochondria, a disrupted mitochondrial structure, loss of the layered structure of the rough endoplasmic reticulum, plasma membrane fragmentation and dissociation of the ribosomes from the endoplasmic reticulum (Fig. 3D).

### Sustained expression of STAT-3 and the expression of MMP-10, VEGF, KDR, HIF-1α, bFGF and IL-10

Expression of STAT-3 and p-STAT-3 increased marginally in early carcinogenesis (4–8 weeks), while in middle- and late-stage (post-12 weeks) carcinogenesis, the expression gradually increased. A significant difference was identified (P<0.01) when compared with the normal group (week 0). The expression of MMP-10, VEGF, KDR, HIF-1α, bFGF and IL-10 was consistent with the changes in the expression of STAT-3 and p-STAT-3.

**Figure 4:**
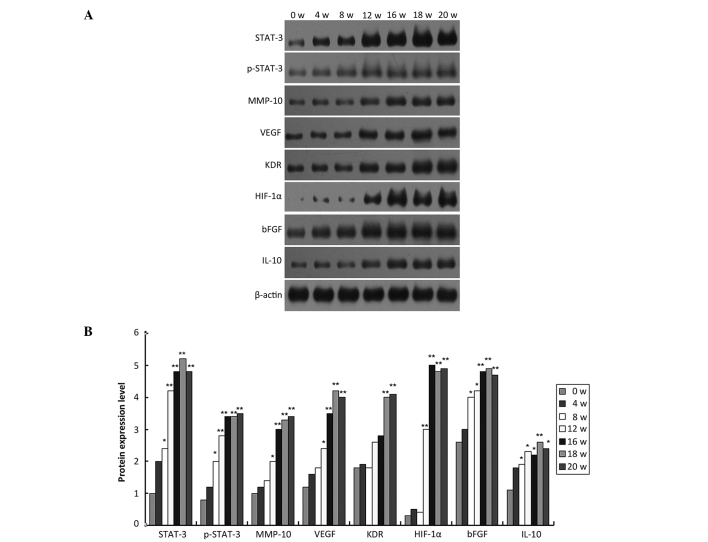
(A) Western blotting detected the expression of STAT-3, p-STAT-3, MMP-10, VEGF, KDR, HIF-1α, bFGF and IL-10 in rat liver tissue of diethylnitrosamine (DEN)-treated rats. (B) The intensity of the STAT-3, p-STAT-3, MMP-10, VEGF, KDR, HIF-1α, bFGF and IL-10 protein bands was determined and normalized against β-actin using the ultraviolet crosslinkers imager, and then plotted (^*^P<0.05, ^**^P<0.01 vs. control group). p-STAT-3, phosphorylated signal transducer and activator of transcription-3; MMP10, matrix metalloproteinase-10; VEGF, vascular endothelial growth factor; KDR, kinase insert domain receptor; bFGF, basic fibroblast growth factor; HIF-1α, hypoxia inducible factor-1α; IL-10, interleukin-10.

**Figure 5:**
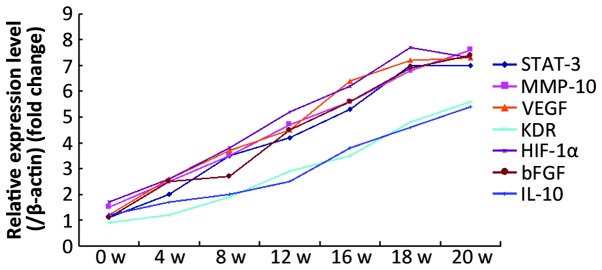
Quantitative polymerase chain reaction (qPCR) analysis of the expression of genes involved in the STAT-3 signal transduction pathway in diethylnitrosamine (DEN)-induced rat liver cancer tissue. STAT-3, signal transducer and activator of transcription-3; MMP10, matrix metalloproteinase-10; VEGF, vascular endothelial growth factor; KDR, kinase insert domain receptor; bFGF, basic fibroblast growth factor; HIF-1α, hypoxia inducible factor-1α; IL-10, interleukin-10.

The western blotting results are shown in Fig. 4A and B. Correlation analysis revealed that changes in the expression of the STAT-3 and p-STAT-3 proteins positively correlated with the expression of the MMP-10, VEGF, KDR, HIF-1α, bFGF and IL-10 proteins (P<0.001) (Table II). qPCR was used to assess the RNA levels of the genes encoding the STAT-3, p-STAT-3, MMP-10, VEGF, KDR, HIF-1α, bFGF and IL-10 proteins. The results showed that the changes in the RNA levels of MMP-10, VEGF, KDR, HIF-1α, bFGF, IL-10 and p-STAT-3 correlated with the protein expression results (Fig. 5; Table III).

## Discussion

Human hepatoma develops as a multi-stage process whereby damage due to hepatitis B or C viral infection causes chronic hepatitis or cirrhosis and then adenomatous hyperplasia nodules form. Early hepatocellular carcinoma (HCC) develops, prior to advanced HCC and HCC metastasis. This multi-stage occurrence and developmental model has been confirmed by pathological analysis and clinical cases (7). In the present study, a modified intermittent administration method developed by Zhang *et al* (8) was used to successfully induce the hepatoma model in Wistar rats. The model is simple, with a brief tumorigenic cycle, and the pathological process follows the general development of human liver cancer (6). The results of the present study were similar to those aforementioned, indicating that this model is stable and has reproducibility.

Tumorigenesis and development are closely associated with signal transduction, and the significant components in signal transduction are the transcription factors, which regulate the expression of oncogenes and thus, indirectly regulate the downstream genes associated with tumor development and diffusion (9,10). STAT-3 is a transcription factor responsive to a variety of cytokines and growth factors via the JAK-STAT signal transduction pathway, which is important in tumor invasion, metastasis, angiogenesis and tumor immune escape (11). The tumor cells must penetrate the barrier formed by the basement membrane and ECM during invasion and metastasis, and damage to the integrity of the basement membrane is a sign of the beginning of malignant tumor invasion. MMPs are able to degrade the majority of the proteins in the basement membrane and ECM. Furthermore, a number of studies (12–14) have found that MMP-10 is expressed in human glioblastoma, and oral, esophageal, stomach, colon, colorectal and liver cancer, as well as other malignant cells. MMP-10 degrades ECM components, including collagen III, collagen IV, gelatin, casein, laminin and proteoglycan elastic hard protein, which is considered to be an important activating factor in human tumor cells that may be activated in other pro-MMPs. In addition, MMP-10 and other activated MMPs may disrupt the basement membrane, providing the necessary conditions for the invasion and metastasis of tumor cells through the vasculature. In the present study, in the DEN-treated rat liver tissue, the expression of MMP-10 increased in a time-dependent manner and degraded the proteins in the basement membrane and ECM, allowing the damaged liver cells to pass through this basement membrane, resulting in the overgrowth of liver cells and finally, carcinogenesis.

Tumor angiogenesis is the basis of tumor cell invasion and metastasis. VEGF is the predominant factor that acts directly to stimulate tumor angiogenesis, and the persistent activation of STAT-3 induces VEGF expression, resulting in tumor angiogenesis (15). VEGF binds to VEGF receptors (VEGFRs), including VEGFR-1 (Flt-1), VEGFR-2 (KDR), VEGFR-3 (Flt-4). KDR is the predominant functional receptor for VEGF. bFGF is another significant pro-angiogenic factor. Tumor cells produce bFGF, and may also induce endothelial cells to produce bFGF, thereby stimulating angiogenesis (16). HIF-1α is a transcription factor that responds to hypoxia. In the present study, DEN treatment was found to promote the expression of HIF-1α in the rat liver tissue, as it bound to the regulatory sequences of the VEGF and bFGF genes, resulting in the increased transcription of VEGF and bFGF, thereby enabling the increased expression of KDR.

Tumor cells use immune suppression and immune tolerance mechanisms to evade surveillance and destruction by the immune system, and promote their own migration and invasion. A previous study (17) demonstrated that STAT-3, by affecting the differentiation and maturation of dendritic cells (DCs), interfered with immune recognition by T cells and thus, the immune system became tolerant of the tumor cells. The results of the present study revealed that activated STAT-3 stimulated the injured liver cells to secrete VEGF and IL-10 to affect the differentiation and maturation of DCs, and thereby inhibited the immune response, allowing the tumor cells to escape it.

In conclusion, the expression of STAT-3, p-STAT-3, MMP-10, VEGF, KDR, HIF-1α, bFGF and IL-10 was investigated in the DEN-induced rat liver cancer model at various stages of carcinogenesis, which confirmed that tumor metastasis, invasion, angiogenesis and immune escape are associated with the sustained activation of STAT-3, thus providing experimental data to support the use of STAT-3 to inhibit HCC.

## Figures and Tables

**Table I:** Primers used in the present study.

Target gene	Sequence (5′-3′)	Product length, bp
β-actin	F: GCAGAAGGAGATTACTGCCCT R: GCTGATCCACATCTGCTGGAA	136
STAT-3	F: CAGCCTGTCGCAGAGTTCA R: GGAGATCACCACAACTGGCA	190
MMP10	F: GGCCCACTCTTCCTTCAGAC R:GAGTGTGGATCCCCTTTGGG	138
VEGF	F: CAAACCTCACCAAAGCCAGC R: GCGCTTTCGTTTTTGACCCT	139
KDR	F: TGGGCAGTCAAGTCCGAATC R: GTTGGTGAGGATGACCGTGT	176
bFGF	F: GCCAACCGGTACCTTGCTAT R: GTCCCGTTTTGGATCCGAGT	187
HIF-1α	F: GCCTTAACCTGTCTGCCACT R: GCTGCTTGAAAAAGGGAGCC	133
IL-10	F: CAGAGAAGCATGGCCCAGAA R: GCTCCACTGCCTTGCTCTTA	129

STAT-3, signal transducer and activator of transcription-3; MMP10, matrix metalloproteinase-10; VEGF, vascular endothelial growth factor; KDR, kinase insert domain receptor; bFGF, basic fibroblast growth factor; HIF-1α, hypoxia inducible factor-1α; IL-10, interleukin-10.

**Table II:** Correlation analysis of STAT-3 and p-STAT-3 protein expression and the expression of the MMP-10, VEGF, KDR, HIF-1α, bFGF and IL-10 proteins.

Protein	STAT-3 (r-value)	P-value	p-STAT-3 (r)	P-value
MMP10	0.820	<0.01	0.969	<0.001
VEGF	0.825	<0.01	0.979	<0.001
KDR	0.738	<0.01	0.950	<0.001
bFGF	0.675	<0.05	0.849	<0.01
HIF-1α	0.752	<0.05	0.916	<0.01
IL-10	−0.748	<0.05	−0.935	<0.001

p-STAT-3, phosphorylated signal transducer and activator of transcription-3; MMP10, matrix metalloproteinase-10; VEGF, vascular endothelial growth factor; KDR, kinase insert domain receptor; bFGF, basic fibroblast growth factor; HIF-1α, hypoxia inducible factor-1α; IL-10, interleukin-10.

**Table III:** Correlation analysis of mRNA levels, comparing STAT-3 with MMP-10, VEGF, KDR, HIF-1α, bFGF and IL-10.

Name	STAT-3 (r-value)	P-value
MMP10	0.990	<0.001
VEGF	0.985	<0.001
KDR	0.994	<0.001
bFGF	0.991	<0.001
HIF-1α	0.984	<0.001
IL-10	0.978	<0.001

STAT-3, signal transducer and activator of transcription-3; MMP-10, matrix metalloproteinase-10 ;VEGF, vascular endothelial growth factor; KDR, kinase insert domain receptor; HIF-1α, hypoxia inducible factor-1α; bFGF, basic fibroblast growth factor; IL-10, interleukin-10; MAPK, mitogen-associated protein kinase.
